# New susceptibility loci for cutaneous melanoma risk and progression revealed using a porcine model

**DOI:** 10.18632/oncotarget.25455

**Published:** 2018-06-12

**Authors:** Emmanuelle Bourneuf, Jordi Estellé, Amandine Blin, Françoise Créchet, Maria del Pilar Schneider, Hélène Gilbert, Myriam Brossard, Amaury Vaysse, Mark Lathrop, Silvia Vincent-Naulleau, Florence Demenais

**Affiliations:** ^1^ CEA, DRF/iRCM/SREIT/LREG, Jouy-en-Josas, France; ^2^ GABI, INRA, AgroParisTech, Université Paris-Saclay, Jouy-en-Josas, France; ^3^ INSERM, UMR-946, Genetic Variation and Human Diseases Unit, Paris, France; ^4^ Institut Universitaire d’Hématologie, Université Paris Diderot, Sorbonne Paris Cité, Paris, France; ^5^ Outils et Méthodes de la Systématique Intégrative, OMSI–UMS 2700, CNRS MNHN, Muséum National d’Histoire Naturelle, Paris, France; ^6^ GenPhyse, INRA, Université de Toulouse, INPT, ENVT, Castanet Tolosan, France; ^7^ McGill University and Genome Québec Innovation Centre, Montréal, Québec, Canada; ^8^ Present address: Ipsen Innovation, Les Ulis, France

**Keywords:** melanoma, biomedical model, pig, GWAS, comparative genomics

## Abstract

Despite major advances, it is estimated that a large part of melanoma predisposing genes remains to be discovered. Animal models of spontaneous diseases are valuable tools and experimental crosses can be used to identify and fine-map new susceptibility loci associated with melanoma. We performed a Genome-Wide Association Study (GWAS) of melanoma occurrence and progression (clinical ulceration and presence of metastasis) in a porcine model of spontaneous melanoma, the MeLiM pig. Five loci on chromosomes 2, 5, 7, 8 and 16 showed genome-wide significant associations (*p* < 5 × 10^–6^) with either one of these phenotypes. Suggestive associations (*p* < 5 × 10^–5^) were also found at 16 additional loci. Moreover, comparison of the porcine results to those reported by human melanoma GWAS indicated shared association signals notably at *CDKAL1* and *TERT* loci but also nearby *CCND1*, FTO, *PLA2G6* and TMEM*38B-RAD23B* loci. Extensive search of the literature revealed a potential key role of genes at the identified porcine loci in tumor invasion (*DST*, PLEKHA5, *CBY1*, *LIMK2* and *ETV5*) and immune response modulation (*ETV5*, *HERC3* and *DICER1*) of the progression phenotypes. These biological processes are consistent with the clinico-pathological features of MeLiM tumors and can open new routes for future melanoma research in humans.

## INTRODUCTION

Cutaneous melanoma is the most deadly skin cancer and has shown a growing incidence worldwide in the last decades [1]. Melanoma is a consequence of the malignant transformation of melanocytes, the pigment-producing cells in vertebrates, and eventually undergoes a metastatic dissemination in draining lymph nodes and inner organs. The only successful treatment so far is *in situ* tumor resection, as classical therapies as chemo-, radio- or immunotherapy are not successful once the tumoral cells have spread away from the primary tumor. However, recent advances in immunotherapy have shown promising results [2].

In humans, cutaneous melanoma results from a major environmental factor (sun exposure), and multiple genetic factors, as first suggested by a family history of melanoma reported in about 10% of the cases [3]. The complex etiology and genetic heterogeneity of this disease makes the discovery of susceptibility genes a challenging task. Two high-penetrance genes, *CDKN2A* and *CDK4*, both regulators of the cell cycle, have been first evidenced in around 20% of the families predisposed to melanoma [4–7]. Rare mutations in other genes, including *POT1*, *TERF2IP* and *ACD*, have been recently identified by whole-exome sequencing in melanoma families [8–10]. The three latter genes are involved in telomere biology together with *TERT* which was also found to predispose to melanoma [11, 12]. Low frequency variants conferring moderate risk of melanoma were identified in two genes, *MC1R* and *MITF*, which play a key role in melanocyte biology and pigment synthesis regulation [13, 14]. Recently, genome-wide association studies have highlighted common genetic variants associated with melanoma risk, at loci containing genes involved in pigmentation and naevus count (e.g. *TYR*, *SLC45A2*, *ASIP*, *PLA2G6*), and DNA repair genes (*PARP1*, *ATM* see [15, 16] for reviews).

Despite these recent breakthroughs, well-suited animal models are still needed to help finding new genes or pathways whose modifications could alter the melanocyte biology and lead to malignant transformation. In the case of melanoma predisposition, only a few species show development of tumors spontaneously and at early onset, with a minimal environmental influence and availability of appropriate genomic tools. These conditions are fulfilled by swine models where melanomas appear spontaneously around birth and where environment can be controlled to a large extent. Three lines of such pigs exist, the Sinclair pig maintained in the US, the Munich Miniature Swine Troll in Germany, and the Melanoma-bearing Libechov minipig (or MeLiM pig), with two herds in France and Czech Republic. The MeLiM pigs develop melanomas spontaneously in the first weeks of life and a careful clinical and histopathological inspection of the lesions has confirmed the relevance of the pig as a model for human melanoma [17]. Additionally, the tumors, even at a metastatic stage, undergo a complete and spontaneous regression after a few months, likely due to a cell cycle arrest of the melanoma cells, followed by an immune system intervention [18].

To gain insight into the genetic component of melanoma occurrence and progression in the MeLiM model, a genome-wide linkage scan was performed to identify quantitative trait loci (QTLs) responsible for melanoma development and sub-phenotypes such as tumor ulceration, skin invasion and metastasis. A backcross pedigree was set up by crossing MeLiM affected pigs with Duroc healthy animals [19, 20], and a fine phenotyping of several melanoma related traits was carried out in 331 animals. The linkage analysis highlighted genomic regions in pigs that were comparable to human loci, such as the orthologue of the HSA 9p21 locus, known to carry both known and uncharacterized melanoma-predisposing genes in humans. However, *CDKN2A* was discarded as a predisposing gene in our model [21], so this linkage signal could be the result of other predisposing gene(s) in the orthologous region of HSA9p21. This study also detected a specific allele of porcine *MC1R* (MC1R^*^2) segregating in the MeLiM herd [20]. This allele carries a coding variation equivalent to the *Sombre* mutation in the mouse (Leu102Pro in pigs, corresponding to Leu98Pro in mice [22]). We therefore hypothesized that this variant, if leading to a constitutive MC1R receptor as seen with the *Sombre* mutant, could influence melanoma penetrance and worsen the phenotype of animals carrying the MC1R^*^2 allele. Also, the most significant linkage peak observed in the genome-wide linkage scan was located on SSC13, at about 77Mb according to the version 10.2 of the porcine genome [20]. The *MITF* gene was located in this QTL interval, but was discarded as a susceptibility gene, even though its involvement in porcine melanoma tumors makes no doubt [23]. Although this first attempt to decipher melanoma predisposition in the porcine model yielded interesting results, the experimental design used a low-density marker panel, with an average of one marker every 20cM, thus preventing a fine-mapping of the linked regions.

In this study, we report the results of a genome-wide association study of melanoma occurrence and progression performed in 190 members of the MeLiM x Duroc pedigree using the porcine 60 K SNP chip [24] (Porcine SNP60 Beadchip, Illumina). The annotation of the GWAS peaks led to the identification of new regions harbouring potential candidate genes for porcine melanoma predisposition. In addition, a comparative genomics approach showed that several melanoma-associated loci, reported by human GWAS [12, 25–34], harbored association signals in the MeliM model, confirming the validity of this model for investigating the genetic component of human melanoma.

## RESULTS

**Figure 1:**
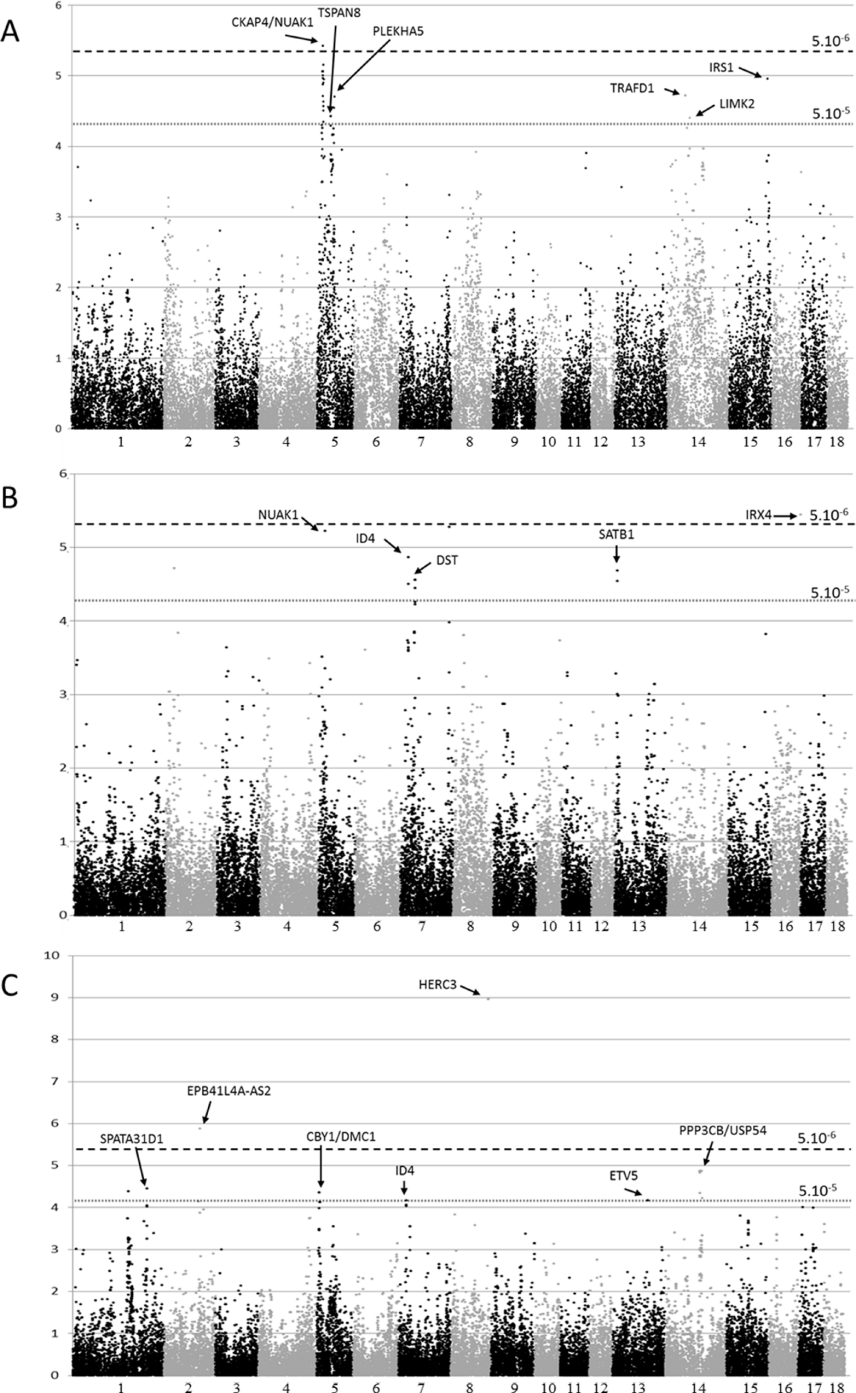
Manhattan plots depicting the results obtained by a genome-wide association analysis of three melanoma phenotypes using the mixed model (**A**) Melanoma occurrence, (**B**) Clinical ulceration, (**C**) Metastasis. The x axis represents chromosomal location and the y axis represents -log_10_ (*p-*value) for the test of association between each SNP and phenotype. The horizontal dashed lines correspond to –log_10_ (*p-*values) of the genome-wide significance threshold of 5 × 10^–6^ and suggestive threshold of 5 × 10^–5^.

**Figure 2:**
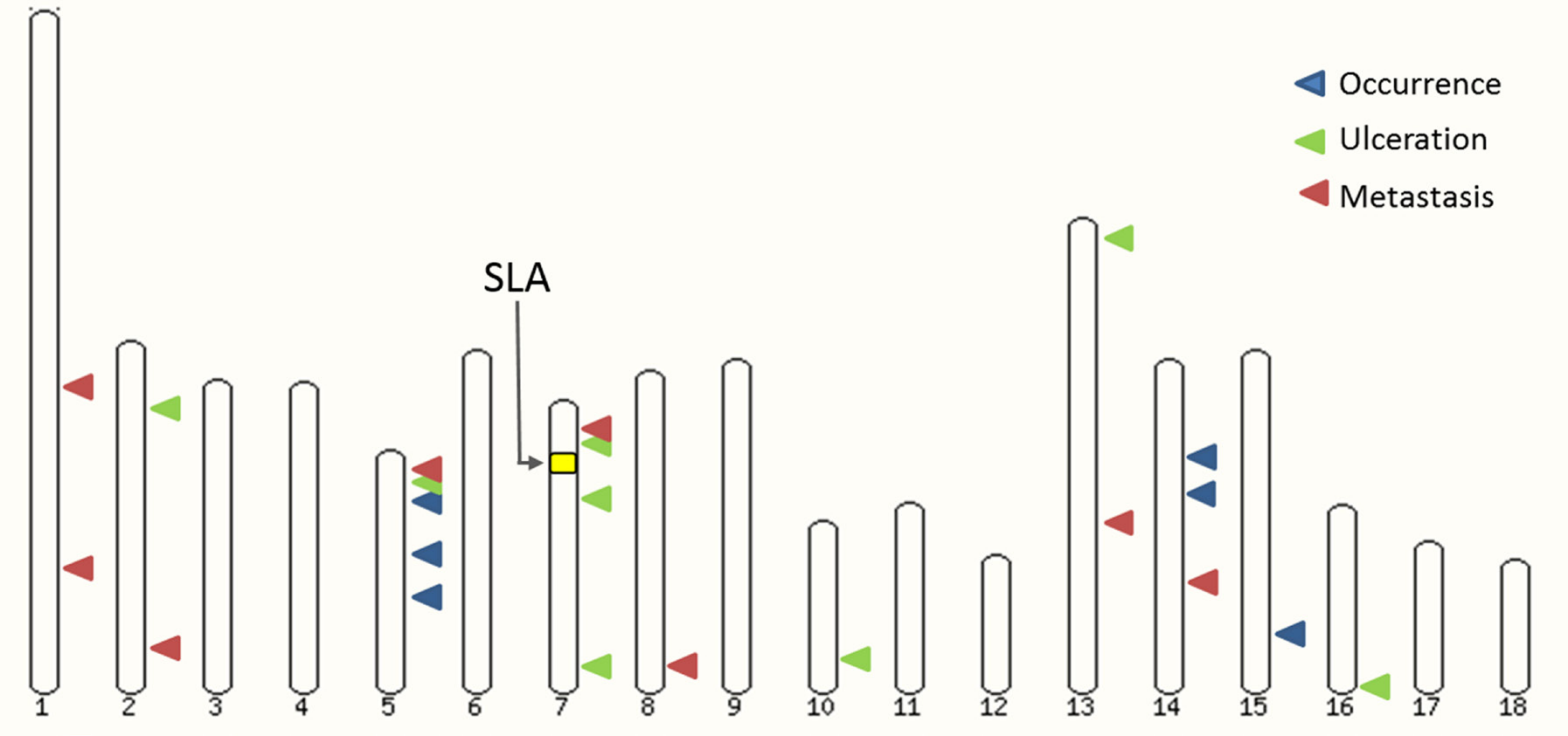
A MeLiM Pig karyotype highlighting melanoma-associated regions on different autosomes

In order to unravel melanoma susceptibility in the MeLiM model, genome-wide association analyses were performed using two statistical methods, the mixed model as implemented in QxPAK [35] and the Fisher’s exact test. Similar results were obtained with these two methods. The associated regions obtained with the mixed model approach are summarized in Tables 1–3 for melanoma occurrence, clinical tumor ulceration and metastasis, respectively. Manhattan plots for the three phenotypes are shown in Figure 1, QQ-plots in Supplementary Figure 1, and significant loci are depicted on the porcine karyotype on Figure 2. The results generated by the Fisher’s exact test in the regions identified by the mixed model method are presented in Supplementary Tables 1 to 3. The genome-wide significance threshold was determined by using the Meff method [36] which calculates the effective number of independent SNPs tested. A multiple-testing Bonferroni-corrected threshold of 5.10^–6^ was used to define genome-wide significance. We also used a less stringent threshold of 5.10^–5^ to identify suggestive association signals.

**Table 1:** Regions showing association with melanoma occurrence (mixed model test)

SSC	Location (bp)^*^	Region size (kb)	Number of SNPs with *p* < 5.10^−5^	Best SNP and position	MAF	Min *p*-value	SNP annotation	Candidate genes (distance from SNP)
**5**	**12950560–15864572**	**2914**	**22**	**H3GA0015760** **(5:14116142)**	**0.11**	**3.81E-06**	**Intergenic**	**Located between CKAP4 (10 kb) and NUAK1 (98 kb)**
**5**	37633973–38064778	430	5	MARC0071790 (5:37767528)	0.17	3.74E-05	Intergenic	Between PTPRR (121 kb) and TSPAN8 (341 kb)
**5**	55008930–57199368	2190	2	DRGA0005864 (5:57199368)	0.166	1.99E-05	Intronic	PLEKHA5, intron 2
**14**	42057436–42257854	200	2	BGIS0007278 (14:42057436)	0.158	1.91E-05	Synonymous coding	TRAFD1, p.Leu313
**14**	51144139	-	1	DIAS0004694 (14:51144139)	0.163	3.98E-05	Intronic	LIMK2, intron 12
**15**	141923807	-	1	ASGA0071118 (15:141923807)	0.331	1.12E-05	Intergenic	Between NYAP2 (1081 kb) and IRS1 (162 kb)

Association was considered significant at the genome-wide level with a *p-*value < 5.10^–6^ (bold), and suggestive with a *p-*value < 5.10^–5^. ^*^Assembly Sscrofa 10.2, Aug 2011 (database version 89.102).

**Table 2:** Regions showing association with clinical ulceration of tumors (mixed model test)

SSC	Location (bp)^*^	Region size (kb)	Number of SNPs	Best SNP	MAF	Min *p*-value	SNP annotation	Candidate genes (distance from SNP)
**2**	21891536	-	1	MARC0015434 (2:21891536)	0.226	1.92E-05	Intergenic	Gene desert
**5**	14863057	-	1	ALGA0030768 (5:14863057)	0.191	6.05E-06	Intergenic	Located between CCNT1 and NUAK1
**7**	16354696–16407894	53	2	ASGA0031451 (7:16354696)	0.482	1.37E-05	Intergenic	Located between ID4 (171 kb) and MBOAT1 (74 kb)
**7**	33574487–33720562	146	3	ALGA0040113 (7:33649465)	0.361	2.75E-05	Intronic	DST, intron 53
**7**	123301941	-	1	ALGA0045159 (7:123301941)	0.442	5.25E-06	Intergenic	Located between GSC (68 kb) and DICER1 (322 kb)
**10**	67879073	-	1	ALGA0115327 (10:67879073)	0.167	2.86E-05	Intergenic	Gene desert
**13**	6211135–6304167	93	2	DRGA0011892 (13:6211135)	0.132	2.07E-05	Intergenic	Located between SATB1 (95 kb) and KCNH8 (1133 kb)
**16**	**85304231**	**-**	**1**	**ASGA0074817** **(16:85304231)**	**0.37**	**3.62E-06**	**Intergenic**	**Located between IRX2 (406 kb) and IRX4 (254 kb)**

Association was considered significant at the genome-wide level if with a *p-*value < 5.10^–6^ (bold), and suggestive with a *p-*value < 5.10^–5^. ^*^Assembly Sscrofa 10.2, Aug 2011 (database version 89.102).

**Table 3:** Regions showing association with metastasis of tumors (mixed model test)

SSC	Location (bp)^*^	Region size (kb)	Number of SNPs	Best SNP	MAF	Min *p*-value	SNP annotation	Candidate genes
**1**	192923645	-	1	DRGA0001676 (1:192923645)	0.281	4.14E-05	Intergenic	Gene desert
**1**	262085978	-	1	ASGA0006090 (1:262085978)	0.414	3.58E-05	Synonymous coding	SPATA31D1, Lys1054
**2**	**121833541**	**-**	**1**	**M1GA0003057** **(2:121833541)**	**0.095**	**1.31E-06**	**Non coding transcript variant**	**EPB41L4A-AS2**
**5**	6499866–6619661	120	2	ALGA0030187 (5:6619661)	0.263	4.44E-05	Intergenic	Located between CBY1 (17 kb) and DMC1 (92 kb)
**8**	**139895592–139896949**	**1,3**	**2**	**ALGA0114256** **(8:139895592)**	**0.139**	**1.09E-09**	**Intergenic**	**Located between HERC3** **(17 kb) and PIGY (45 kb)**
**13**	133415925–134127955	712	2	MARC0004732 (13:133415925)	0.172	4.03E-05	Non synonymous coding (predicted)	ETV5, Tyr271Cys
**14**	81218453–85991839	4773	10	ASGA0064587 (14:82432562)	0.08	1.37E-05	Intergenic	Located between PPP3CB (1 kb) and USP54 (0,9 kb)

Association was considered significant at the genome-wide level if with a *p-*value < 5.10^–6^ (bold), and suggestive with a *p-*value < 5.10^–5^. ^*^Assembly Sscrofa 10.2, Aug 2011 (database version 89.102).

### Regions showing association with melanoma occurrence

Three chromosomes harbored regions that were associated with melanoma occurrence (defined by no lesions versus confirmed malignant lesions) in this population of the MeLiM model (Table 1). However, SSC5 and SSC14 contained three and two distinct regions, respectively, leading to a total of six regions including 1 to 22 SNPs with *p* < 5.10^–5^. Genome-wide significance was reached by one region, while the other five ones achieved suggestive levels, and four of the six regions were confirmed by the Fisher’s exact test (Supplementary Table 1).

The first region on SSC5, extending from 12.9 to 18 Mb (Assembly Sscrofa 10.2, Aug 2011), was the most significant one, with a genome-wide significant *p-*value for SNP H3GA0015760 (*p* = 3.81 × 10^–6^), and comprised 21 additional SNPs with a *p-*value < 5.10^–5^. The SNP with the lowest *p-*value was located between genes *CKAP4* (cytoskeleton associated protein 4) and *NUAK1* (AMPK-Related Protein Kinase 5). CKAP4 is a dickkopf1 receptor involved in tumor progression *in vitro* and *in vivo* [37], while NUAK1 promotes survival and invasion of cancer cells [38]. The second region of SSC5 spanned 430 kb between 37.6 and 38.1 Mb, with the strongest association signal (*p* = 3.7 × 10^–5^) located between *PTPRR* (Protein Tyrosine Phosphatase, Receptor Type R), a negative regulator of the MAPK pathway [39], and *TSPAN8* (Tetraspanin 8), a key regulator of melanoma invasion [40, 41]. The third SSC5 region extended over 2 Mb (between 55 and 57 Mb), with the best SNP located within the *PLEKHA5* (Pleckstrin Homology Domain Containing A5) gene (DRGA0005864, *p-*value = 1.99 × 10^–5^), recently described as a mediator of distant melanoma metastasis in the brain [42].

On SSC14, a synonymous coding SNP in the *TRAFD1* (TRAF-Type Zinc Finger Domain Containing 1) gene reached a *p-*value of 1.91 × 10^–5^, although no direct link exists between melanoma occurrence and TRAFD1 function, a regulator of toll-like receptor signaling [43]. The second associated region on SSC14 was evidenced by one intronic SNP, located in *LIMK2* (LIM Domain Kinase 2), a kinase involved in keratinocyte adhesion [44]. Finally, the top SNP of the SSC15 region was located only 162 kb away from *IRS-1* (insulin receptor substrate 1), coding for the protein transmitting signals from the IGF-1 receptor to PI3K/AKT and ERK/MAPK pathways, known to be involved in melanoma [45].

These association results were then compared to the linkage analysis performed by Du *et al.* [20] on a so-called “synthetic trait”, which corresponded to a continuum of four melanoma occurrence categories (Table 4), thus differing from the current definition of melanoma occurrence that included the extreme classes 1 (no lesions) and 4 (malignant lesions). No significant linkage peak was detected on SSC5 which may be partly due to the poor coverage of SSC5 with microsatellites. However, two regions on SSC14 and SSC15 were also identified by Du *et al.* [20]. The interval on SSC14 was 10% chromosome-wide significant, and included the SW1709 microsatellite, located 9 Mb apart from the melanoma-associated SNP DIAS00004694. The linkage signal on SSC15 (5% chromosome wide significant) was represented by SW936 (located at 135 Mb), less than 7Mb apart from the association signal.

**Table 4:** Phenotypes recorded on the pedigree, and distribution of individuals within phenotypic groups

Description of phenotypes		Comments
***Sex*** **Males** **Females**	152 139	An absence of gender effect was shown previously (Du *et al.*, 2007).
***Coat Color*** **Black** **Grey** **Red/Brown**	34 101 156	Coat color, through MC1R genotype, is associated with melanoma occurrence. Black animals are predisposed to melanoma (Du *et al.*, 2007).
***Melanoma Occurrence*** **No lesion** **Flat lesions** **Raised lesions** **Malignant melanoma**	100 40 105 46	Animals are distributed to the group describing their most advanced lesion. Absence of lesion Flat lesions which were either histologically benign or could not be histologically analyzed because of their large number Pigs bearing small raised lesions with a slow growth phase (SSM or NM-Clark’s levels II–IV) or flat lesions classified as SSM-Clark’s level I; these lesions appear during the first 3 postnatal months One or more large raised or polypoid lesions, often ulcerated and associated with metastasis; these lesions correspond to deeply invasive melanoma (SSM or NM-Clark’s level IV–V) and mostly develop in utero or during the first 3 months of life
***Tumor ulceration*** **Yes** **No** **Undetermined**	34 156 1	Clinical ulceration is reported if it has been observed at least for one lesion per individual. Unaffected animals are not included.
***Metastasis*** **Yes (adenomegaly, visceral)** **No** **Undetermined**	32 157 2	Metastasis is recorded in affected animals, if adenomegaly was detected by palpation or metastasis seen at necropsy. Unaffected animals are not included.

### Association with melanoma progression

The MeLiM model recapitulates several clinical factors that can be scored precisely and accurately used as phenotypes in a GWAS. Thus, a GWAS was also conducted for clinical tumor ulceration and metastasis, defined, respectively, by the detection of lymphadenopathy at palpation of affected animals, and the observation and histological validation of nodal or visceral metastasis.

The genome-wide significant association signal for ulceration was located on SSC16, nearby the *IRX4* gene (Iroquois homeobox gene), that was identified by a GWAS for prostate cancer risk in humans [46]. Interestingly, the *TERT* gene is located only 600 kb away from *IRX4* and is now considered as a major player of melanoma predisposition [47]. Among the seven suggestive regions for ulceration, an interesting one was located on SSC5, nearby *NUAK1* as for melanoma occurrence. However, while the occurrence-associated SNP lies 98 kb upstream of *NUAK1*, the variant associated with ulceration was located 600 kb downstream (ALGA0030768, genome-wide significant *p-*value = 6.05 × 10^–6^). The chromosome 7 harbored three different regions showing suggestive association with the ulceration phenotype. In the first region (SSC7: 16354696-16407894bp), the top SNP ASGA0031451 is located between *ID4* (Inhibitor of DNA Binding 4, HLH Protein) and *MBOAT1* (Membrane Bound O-Acyltransferase Domain Containing 1) and reached a *p-*value of 1.37 × 10^–5^. MBOAT1 is related to lipid biosynthesis [48] and still poorly described, while ID4 role in melanoma is more documented and may take part in phenotypic switch of melanoma cells [49–51]. Interestingly, a suggestive association signal was also obtained between this region and metastasis when analyzing the data with the Fisher’s exact test (data not shown). A second region on SSC7, spanning 146 kb at around 33.5Mb, was also associated to ulceration with the most significant SNP located within the *DST* gene (ALGA0040113, *p-*value = 2.75 × 10^–5^). *DST* codes for the dystonin protein, involved in keratinocyte integrity and mutated in a specific subtype of Epidermolysis Bullosa simplex [52–53]. The third region detected on SSC7 contained one SNP which almost reached the genome-significance level (ALGA0045159, *p-*value = 5.25 × 10^–6^) located between *GSC* (Goosecoid Homeobox), coding for an homeobox protein [54], and *DICER1*, the endoribonuclease involved in miRNA processing machinery. Several groups have explored the association between *DICER1* expression in melanoma tumors and patient survival but obtained conflicting results [55, 56]. On SSC13, two SNPs delineate a region of 93 kb, located less than 100kb away from *SATB1* (SATB Homeobox 1). *SATB1-AS1*, a lncRNA targeting *SATB1*, is located at this locus in humans, but has not been described in pigs yet. *SATB1* regulates chromatin remodelers, and its expression is correlated with proliferation and invasiveness of melanoma cells *in vitro* and *in vivo* [57, 58]. For the other two regions on SSC2 and SSC10 associated with ulceration, the top SNPs were in gene deserts.

Regarding metastasis, we found two genome-wide significant regions on SSC2 and SSC8, and five suggestive signals, on SSC1, SSC5, SSC13 and SSC14. The most significant marker was found on SSC8 (ALGA0114256, *p* = 1.09 × 10^–9^), and comparative genomics indicates that it is located in a sequence predicted to be the first intron of the *HERC3* gene. HERC3 is described as a regulator of immune response, notably through NF-κB [59]. The SNP reaching significance with the mixed model on SSC2 (M1GA0003057) was confirmed by the Fisher’s exact test (Supplementary Table 3). Again, relevant candidate genes at these loci can be identified. Among those, a lncRNA, located in the genome-wide significant SSC2 region, *EPB41L4A-AS2*, was recently described as a potential tumor suppressor in solid tumors [60]. Interestingly, comparative genomics shows that another lncRNA, *PPP3CB-AS1*, is predicted to lie in the locus harboring ASGA0064587, a SNP tagging the 4.7 Mb region on SSC14 associated with metastasis. Also, *PPP3CB-AS1* has been shown to be overexpressed in metastatic lesion of pancreatic cancer compared to primary tumors [61]. The locus defined by two SNPs on SSC5 (at 6.5–.6 Mb), reached a minimal *p-*value of 4.4 × 10^–5^, and included the *CBY1* gene. Remarkably, *CBY* codes for an antagonist of β-catenin, which plays a key role in melanoma tumor, by regulating β-catenin mediated transcriptional activation [62]. Another cross-species comparison between human and porcine genomes indicated that the SNP MARC0004732 on SSC13 (133415925) can be annotated as a non-synonymous coding variant of the *ETV5* (ERM-related molecule) gene. This polymorphism corresponds to a Tyr271Cys modification, and is predicted as a deleterious variant in humans (rs770229110). This SNP has been identified in several catalogues of variation in pig genomes and probably segregates in different populations. In Human, the alternative allele, coding for a cysteine instead of a tyrosine, has only been observed at a frequency <10^–5^ in the ExAc consortium data, corresponding to only one allele in more than 60000 individuals, and has not been related to any pathology so far. ETV5 belongs to the ETS transcription factors family, involved in various processes such as cytokine production or migration of tumor cells [63, 64]. Finally, the association signals in the two regions on SSC1 lie in a gene desert for the first one and near *SPATA31D1* for the second one.

As shown above, the results of the mixed model analysis suggested potential involvement of genes with a role documented in cancer. However, the SNP genotyping chips do not cover all regions of the genome identically, which could bias our interpretation. Thus, we surveyed all genes present in the defined intervals or in extended regions within 500 kb of the single-SNP association signals (Supplementary Tables 4–6). Even if most of the genes mentioned above represent excellent functional candidates, one should consider a potential role of other genes in these larger intervals. Complementary studies, such as expression analysis, need to be conducted to determine which candidate genes are the most relevant in our model. For example, *KRAS* (KRAS Proto-Oncogene, GTPase) is also located in the SSC5 interval containing *PLEKHA5* and is an appealing candidate for melanoma development since RAS proteins frequently undergo somatic or even germline mutations in melanoma [65]. However, we did not identify any germline mutation in *KRAS* segregating in the pedigree (data not shown).

### Comparison with melanoma-associated loci in humans

A few genes located nearby the most significantly associated SNPs in this study have been described as involved in melanoma tumor biology (*CKAP4*, *ID4*, *SATB1*, and *PLEKHA5*), but not in melanoma susceptibility. Indeed, when we compared the regions described above with the significant loci reported by human melanoma GWAS, no common locus was clearly identified. Two exceptions might be the *CDKAL1* and *TERT* melanoma risk loci, that lie near the orthologous SCC7 and SSC16 ulceration-associated regions, respectively, as described below.

In order to validate the relevance of the porcine model for analyzing the genetic susceptibility to melanoma in humans, we established a comprehensive list of human loci significantly associated with melanoma risk from GWAS [25–34]. These loci were mapped to the porcine genome and extended by 500 kb on each side of the lead human SNP. Table 5 presents the list of human loci and annotated genes for which pig SNPs at orthologous regions were associated to at least one of the three different phenotypes examined in this study with a *p-*value < 10^–2^. Out of 20 orthologues of human melanoma-associated loci, 12 contained SNPs with *p-*values < 10^–2^ in the MeLiM model, and 6 of them had SNPs reaching a *p-*value < 10^–3^: *CDKAL1* on SSC7, *FTO* on SSC6, *PLA2G6* on SSC5, *TMEM38B-RAD23B* on SSC1 and *SLC45A2* and *TERT* on SSC16. Six extended loci showed *p-*values < 10^–2^ for at least two phenotypes (*CCND1* on SSC2, *CDKAL1* on SSC7, *TERT* on SSC16, *FTO* on SSC6, *PLA2G6* on SSC5 and *RAD23B* on SSC1) while the *CDKAL1* genomic region harbored SNPs with *p-*values < 10^–3^ for the three phenotypes. These results need to be interpreted in the light of the limited number of markers at 1 Mb loci given the low and heterogeneous density of the porcine SNP chips. Indeed, the *CDKAL1* region is only 400 kb from the SSC7 region associated with ulceration at *p* = 1.4 × 10^–5^ and the *TERT* locus is less than 600 kb from the genome-wide significant signal for ulceration on SSC16.

**Table 5:** *p*-values obtained in the swine GWAS for genes associated with human melanoma

Human Gene Name	Associated Phenotypes	Human Locus	Location on pig genome	Nb of SNPs within 1Mb, with a MAF >0,1	Nb of SNPs with a *p-*value <10^–2^	Minimal *p-*value found in the 1Mb interval	SNP ID	Position on pig genome	MAF
***ATM***	Ulceration	11q22.3	9:40925895–40945439	11	1	9.38E-03	ALGA0056075	9: 40925224	0.181
***CCND1***	Tumor occurrence	11q13.3	2: 2342270–2343202^*^	20	1	7.40E-03	INRA0008096	2: 2666056	0.43
	Ulceration			22	1	8.47E-03	ALGA0112306	2: 1868626	0.132
***CDKAL1***	Tumor occurrence	6p22.3	7:16819884–17481027	35	2	7.68E-04	H3GA0020098	7: 16370134	0.389
	Ulceration			35	10	5.48E-04	ASGA0031451	7: 16354696	0.482
	Metastasis			35	1	3.20E-05	H3GA0020098	7: 16370134	0.389
***CLPTM1L-TERT***	Tumor occurrence	5p15.33	16:85904690–85932895	7	1	9.24E-03	H3GA0047553	16: 85723254	0.429
	Ulceration			8	2	7.00E-04			
***FTO***	Ulceration	16q12.2	6: 28248675–28592703^*^	39	5	3.48E-04	ASGA0085552	6: 28115104	0.16
	Metastasis			25	2	1.52E-03			
**Locus *ARNT, SETDB, LASS2***	Ulceration	1q21.3	4:107510690–107636206	30	1	4.67E-03	ALGA0027243	4: 108252729	0.453
***MX2***	Metastasis	21q22.3	13:215028064–215059085	17	2	7.12E-03	ALGA0074008	13: 214911346	0.383
***OBFC1***	Tumor occurrence	10q24.33		17	3	7.49E-03	ASGA0066225	14: 124353052	0.288
***PARP1***	Metastasis	1q42.12	10: 16598158–16636256	28	1	4.09E-03	ASGA0085873	10: 16744185	0.21
***PLA2G6***	Tumor occurrence	22q13.1	5: 6996414–7059756	14	1	7.07E-03	MARC0059533	5: 7491676	0.359
	Metastasis			19	1	4.47E-04			
***SLC45A2***	Ulceration	5p13.3	16:20718194–20758825	34	7	3.84E-04	ASGA0100023	16: 20279413	0.121
***TMEM38-RAD23B***	Tumor occurrence	9q31.2	1:276767826–278948163	51	4	4.12E-03	H3GA0004197	1: 279069362	0.247
	Ulceration			51	5	5.11E-04	INRA0006959	1: 278401825	0.352
	Metastasis			51	1	3.53E-03	H3GA0004197	1: 279069362	0.247

approx. location*

Data correspond to the Fisher’s exact test on the 3 phenotypes Tumor occurrence, ulceration, and metastasis). *Approximate location estimated by comparative genomics between human and pig genomes.

Finally, in order to facilitate the comparison between the pig model and human disease, the coordinates of each significant peak obtained with at least one of the 3 phenotypes in pig were compared to the human genome to determine corresponding borders, on physical and cytogenetic maps (Supplementary Table 7).

## DISCUSSION

Here we report the first genome-wide association study performed in a swine model for human cutaneous melanoma, the MeLiM pig. In previous studies, the major predisposition genes for human melanoma were discarded: for example, *CDKN2A* was excluded thanks to an association and a haplotype analysis [21]. *CDK4* alleles did not segregate with melanoma in a reference population [19], and lastly an association analysis showed that *MITF* could not be related to melanoma occurrence in the MeLiM model [23]. This study used the power of a backcross pedigree to identify 21 genomic regions associated with melanoma occurrence and/or progression phenotypes, *ie* clinical ulceration and presence of local and/or distant metastasis (superficial lymphadenopathy *vs* visceral metastasis). Moreover, four of these regions reached genome-wide significance.

Association signals were often observed over broad chromosomal regions, which is rarely the case in GWAS performed in samples of unrelated cases and controls. All animals in this study were members of the same pedigree so that many variants were carried by long-range haplotypes, as reflected by similar *p-*values shared by relatively distant markers. This experimental design makes it difficult to fine-map the identified regions but has the advantage of partly circumventing the issue of the low density of pig SNP arrays by increasing the linkage disequilibrium between markers and thus the power of association studies.

Although a large pedigree structure is also well suited for linkage analysis, a few discrepancies were observed between the results of the previous linkage-based study of Du *et al.* [20] and the present association study applied to animals from the same cross. These differences may be due to differences in the type of information used by these two approaches (*ie* familial transmission of risk haplotypes with disease in linkage studies and linkage disequilibrium mapping between markers and putative disease variants in association studies), the set of markers used (153 microsatellite markers for linkage analysis and 60 K SNPs for GWAS) and the phenotype definition. Regarding melanoma occurrence, a continuum of four classes was used in the linkage analysis versus two extreme classes in the association analysis. Although the latter phenotype allowed reducing phenotypic heterogeneity by eliminating individuals carrying tumors with an uncertain malignant status, this might have decreased power by reducing the sample size. The mixed model approach used herein proved to be relevant and robust, as signals were confirmed by the more conservative Fisher’s exact test.

### SSC5 and SSC7 harbor broad regions associated with pig melanoma occurrence and progression

The SSC5 chromosome harbored four regions associated with melanoma occurrence and/or progression phenotypes. One of these regions, that extended over 3 Mb, was associated with both melanoma occurrence and ulceration and several lines of evidence make *NUAK1* the best candidate gene in that region. NUAK1 is an AMPK-related kinase that favors melanoma invasion *in vitro* [66]. NUAK1 is phosphorylated by LKB1 (Liver Kinase B1), which carries somatic mutations in some cases of cutaneous melanoma [67, 68]. Also, the LKB1-AMPK pathway, including NUAK1 has a prominent role in growth control and tumor suppression [69]. Thus further experiments are needed to understand how NUAK1 could affect melanoma growth in pigs.

The SSC7 chromosome harbors three regions associated with ulceration. Notably, we found two distinct signals, on either side of the SLA (Swine Leukocyte Antigen) locus (Figure 2). These two regions and the good resolution provided by SNP genotyping chips could explain why a signal was observed near SLA in previous studies in MeLiM [19] and Sinclair [70] models. In the Sinclair model, a two-locus model was proposed, where an unknown major locus was modified by a specific SLA haplotype while in the previous MeLiM X Duroc cross study, a broad Duroc SLA haplotype was found associated with melanoma occurrence. The best SNP in the first SSC7 associated region is located near *ID4*, for which overexpression has been shown to enhance pigmentation of melanoma cells and induce tumor necrosis [49]. This feature is of particular interest for our model, since the MeLiM tumors are highly pigmented and spontaneously regress after a few weeks of age. Yet, *CDKAL1* resides only 650kb away from the best SNP ASGA0031451, and was significantly associated with human melanoma in a large-scale GWAS [71]. A lower SNP coverage in pigs could explain that despite a less significant *p-*value, *CDKAL1* could be a relevant candidate for porcine melanoma, accounting for the SCC7 signal, although more investigation is needed.

The second SSC7 region located downstream of the SLA locus, encompasses the *DST* gene, coding for the BPAG1-e protein. This protein is a member of the plakin family [71] and plays a major role in keratinocyte adhesion and mobility in the epidermis [53]. In addition, mutations in the corresponding gene *DST* have been identified in epidermolysis bullosa simplex, highlighting a key role in skin homeostasis [52]. Finally, *DST* has been identified as a downregulated transcript in ulcerated tumors of melanoma patients [72]. Thus a link between BPAG1 and melanoma ulceration, which is a disruption of the epidermis, seems relevant and needs to be further explored. Also, melanoma ulceration is considered as one of the predictors of poor prognosis in patients, hence more precise molecular characterization would be beneficial, notably by focusing on germline variants influencing melanoma ulceration.

A single significant SNP defines the third region on SSC7 and is located between *GSC* and *DICER1* genes. Both genes could represent appealing candidates for melanoma and would deserve further description. For example, as detailed thereafter, DICER1 is currently considered as a pivotal actor in melanoma biology [73].

### Three hallmarks of cancer particularly affected in the MeLiM model

For melanoma occurrence and metastasis, our analysis identified a potential contribution of four genes described as modulators of local invasion or metastatic process. *TSPAN8* (located on SSC5) has been thoroughly described in melanoma recently [40, 41], and its elevated expression in tumor cells favors early invasion but has no effect on survival or proliferation. In contrast, Jilaveanu and colleagues [42] showed that *PLEKHA5* (also on SSC5) was involved in melanoma cells survival and “brain homing” of metastatic cells through extravasation, but the exact mechanisms remain unknown. CBY1 prevents the transcriptional activity of beta-catenin, which alteration is a key element in radial growth phase of melanoma [74]. Although it has not been investigated directly in melanoma cells, we can speculate a potential activity of CBY1 in this neoplasm. Also, in colorectal cancer cells, *CBY1* knockdown has been shown to promote mesenchymal to epithelial transition [75]. In addition, the top SNP on SSC14 is intronic to *LIMK2*, a kinase of the actin signaling pathway. LIMK2 promotes actin stabilization and inhibits terminal differentiation of keratinocytes of the basal layer of the epidermis [44]. Another interesting finding is the association between a predicted non-synonymous SNP in *ETV5* coding sequence and presence of metastasis in the model. Indeed, ETV5 is a transcription factor, downstream target of Met signaling, and involved in migration and invasion in Met-addicted solid tumors [64]. Also, a study investigating the targets of ETV5 in an endometrial carcinoma model has evidenced a regulation of adhesion molecules and epithelial-mesenchymal transition (EMT) genes [76]. Although the status of MeLiM tumors regarding classical oncogenes has not been explored yet, this function in invasion is worth considering. Overall, the documented functions of many genes identified in our study relate to epidermis structure modification and/or invasion to distant locations.

A second interesting functional class well-represented in this study concerns actors of the immune response. Indeed, an appealing genetic model in the MeLiM pig would be a dual action of germline variants on melanoma occurrence and regression, since all animals affected regress completely and spontaneously without intervention. Tumor regression could be mediated by more efficient immune actors and/or an increased immunogenicity of melanoma cells. Thus, genetic variants modulating the effect of genes relevant for both tumor development and immune response would be of great interest in the MeLiM model.

In addition to the already mentioned role in MET and MAPK pathways [64, 77], ETV5 transcription factor regulates the production of IL-10 in Th2 cells [78], and IL-9 transcription in Th9 cells [79]. IL-10 is expressed in immune cells present in melanomas (melanophages, lymphocytes) and also by tumor cells as invasion proceeds [80], fostering an immunosuppressive environment favorable for tumor development. Conversely, IL-9 and Th9 cells are rather associated with an anti-tumor immunity [81]. Finally, Jojic *et al.* [63] demonstrated that ETV5 is a γδT-cell differentiation regulator in mouse. Pig is a “high γδ species”, with abundant circulating γδT cells [82]. Whether ETV5 modulates their differentiation also in pig or it affects γδ-mediated immune response in porcine melanoma remains undetermined. The SSC8 region associated with metastasis harbors *HERC3*, reported for its function in the modulation of NF-κB-initiated inflammation [59]. DICER1 (SSC7), associated with ulceration is a key player of miRNA synthesis and responsible for the DICER1 syndrome, a condition that increases the risk of developing various types of tumours in families of patients. In their article, Hoffend and colleagues [73] report an unexpected role for DICER in inducing anti-tumor immunity. Indeed, *DICER* knocked-down tumors had a slower growth than controls, due to a more immunogenic phenotype of cells. MHC Class I molecules were more expressed at the surface of these cells, and inhibitors of immune response such as PD-L1 were repressed. Finally, the authors show that this difference relies on an enhanced cytotoxicity of CD8+ T cells.

To finish with, one of the essential characteristics of a tumor is a facilitated proliferation, most familial melanomas being mutated in *CDKN2A* or *CDK4* thus enhancing cell divisions. Two genes associated with pig melanoma (*IRS1*, *NUAK1*) were described as potential key players in human melanoma tumors [66, 83] and *EPB41L4A-AS2* showed tumor suppressor features in breast cancer [60].

In conclusion, three main hallmarks of cancer, as described by Hanahan and Weinberg [84] could be involved in the genetic basis of melanoma in the MeLiM model: sustaining proliferative signaling, activating invasion and metastasis, and modulating immune response. This result illustrates the possibility of using such a model to orientate the search for germline variants in human melanoma.

In addition, a preliminary exploration of data by enrichment analysis (Enrichr [85]) and IPA^®^ (Qiagen) suggested a link between a majority of the genes identified herein and TGFβ signaling. For example, ID4 and NUAK1 belong to the TGFβ pathway, while genes like *PTPRR*, *PLEKHA5*, *DICER1*, *SATB1*, *IRS1* and *IRX4* are targets of SMAD proteins, the transcription factors which are downstream effectors of this signaling. The involvement of TGFβ in melanoma has been explored by several groups, and its role on proliferation inhibition has been shown in many studies. Hoek *et al.* [86] have demonstrated that different melanomas show different sensitivities to TGFβ mediated inhibition. Sensitive tumors usually proliferate less but acquire a higher metastatic potential, and exhibit a typical signature of TGFβ downstream target genes. Interestingly, this TGF-β signature was also found in a transcriptomic study performed on tumor regression in the MeLiM [18]. Thus, the impact of TGFβ in the MeLiM model should be investigated, as a modulator/inhibitor of proliferation and invasion.

### Comparative genomics to identify new candidates for human melanoma predisposition

Comparative genomics can be a powerful strategy to evidence disease genes in humans. Even though major melanoma predisposing genes such as *CDKN2A* and *CDK4* have been discarded in the MeLiM model, six loci that have been associated with human melanoma show association signals in pigs. First, the *TMEM38B-RAD23B* locus had several SNPs with a *p*-value < 10^–3^ for the three traits considered here. In humans, the most significant SNPs were located in the intergenic region between *TMEM38B* and *RAD23B*. A more detailed analysis in pigs might help prioritizing the most relevant candidate in humans. For instance, in human, the association with *CCND1* did not reach genome-wide significance requirements in a first GWAS [25] but was definitely demonstrated in a meta-analysis of larger sample size [11]. The association signal found with the porcine *CCND1* locus illustrates the utility of animal models to strengthen human findings. The *FTO* and *PLA2G6* genes, which show significant associations with melanoma in both species, have been more classically studied in the light of metabolism and adiposity traits [26, 87]. However, at least for *FTO*, the association with melanoma was independent of the body mass index association signal. Thus, complex mechanisms related to metabolism might be involved in melanoma development in humans as well as in pigs, highlighting the comparability of the two species in this regard.

With respect to the *TERT* locus, an extensive sequencing of the gene and its promoter in pigs is required to search for mutations similar to the ones observed in melanoma and other human cancers. In addition, the telomerase activity could be evaluated in MeLiM tumors, and relationship with the progression and regression phases as already described. Indeed, in the Sinclair swine, the telomerase activity is lost in regressing tumors, but whether telomere maintenance is causal of regression remains to be established [88]. Moreover, the *CDKAL1* locus also deserves to be further explored.

In addition to these six loci, our association study evidenced novel regions which have not yet been reported in human melanoma. Investigation of the syntenic regions of these loci in human datasets may bring new findings in susceptibility to human melanoma. Furthermore, integration of functional data such as genome-wide expression data to GWAS results might greatly help prioritizing the genes to be explored further.

### Conclusion and perspectives

In conclusion, we report here the first genome-wide association study of cutaneous melanoma in the pig species using the MeLiM model. This study identified new loci that have not yet been found as predisposing to human melanoma. It also showed that a few regions robustly associated with human melanoma might be involved in pig melanoma occurrence and/or progression, highlighting the interest of cross-species comparisons. As a consequence, the MeLiM model represents a useful tool by providing new candidates to be tested in susceptibility to both melanoma occurrence and progression in humans.

## MATERIALS AND METHODS

### Animals and phenotyping

The backcross MeLiM X Duroc pedigree set up to perform a linkage study of melanoma development has already been described [18]. Briefly, 4 MeLiM affected animals were crossed to 5 Duroc pigs and produced a F1 generation. Nine F1 affected animals were backcrossed to 25 Duroc individuals to produce 42 backcross families, for a total number of 331 backcross (BC) pigs. In this study, all F1 and 291 BC animals were phenotyped for melanoma traits, from birth to 3 months of age (Table 4). The first phenotype corresponded to melanoma occurrence, with scores based on the presence of lesions, their histological type and the clinical evolution of the disease. Then, 4 score classes were addressed, from absence of lesion (class 1, *n* = 100), flat lesions (class 2, *n* = 40), raised lesions (Class 3 : Superficial Spreading melanoma (SSM) or Nodular Melanoma (NM) with Clark’s levels II–IV [89] or flat lesions classified as SSM-Clark’s level I, *n* = 105), to malignant melanoma (class 4: large, polypoid lesions, often ulcerated and associated with metastasis, corresponding to deeply invasive melanoma (SSM or NM-Clark’s level IV–V), *n* = 46). Animals bearing malignant melanoma were also scored for clinical ulceration of tumors (presence or absence, *n* = 34 and 156 respectively) and presence and type of metastasis (superficial lymphadenopathy detected by palpation or lymph node and visceral metastasis observed at necropsy and confirmed by histology). Metastasis was observed in 32 animals, while it was absent in the remaining 157 pigs. All procedures involving animals were performed according to the applicable veterinary and ethical rules.

### Genotyping and data QC

DNA samples from 9 F0, 34 F1 and 291 BC animals were obtained from blood lymphocytes after classical DNA extraction methods. A quality control of the samples was performed at CNG (National Center for Genotyping, Evry, France) before genotyping. The samples were genotyped on the porcine SNP60 beadchips v1 (Illumina) and SNP calling was carried out with GenomeStudio^®^ Data Analysis software.

The SNPs were retained for further analysis if their call rate was > 95%, with a Minimum Allele Frequency > 10%. The Hardy Weinberg Equilibrium was not taken into account because this work was performed in closely related individuals and individuals were not mated randomly. The mendelian inheritance within the pedigree was checked with the Pedcheck software [90] and 785 inconsistent markers were removed from the analysis. Finally, 47536 out of 64232 markers were retained for association analysis. More than 90% of the markers were genotyped in all samples, thus none of the 334 samples included in this study was excluded.

### Statistical analysis

The genome-wide association analysis was first performed on the melanoma occurrence phenotype, *ie* class 1 versus class 4 (*n* = 100 and 46 respectively), to avoid a potential bias produced by intermediate benign lesions (Table 4). Second, the association analysis was conducted on progression phenotypes, *ie* clinical ulceration of tumors (*n* = 190) and metastasis (*n* = 189).

The genome wide-association analysis was performed for each SNP individually by using a mixed model that takes into account the family structure and relatedness between animals with the QxPAK software [35]. This model, based on a maximum likelihood approach, is the following:

$${\text{y}}_{\text{i}}={\text{sex}}_{\text{i}}+{\text{color}}_{\text{i}}+{\text{aSNP}}_{\text{i}}+{\text{dSNP}}_{\text{i}}+{\text{u}}_{\text{i}}+{\text{e}}_{\text{i}}\text{,}$$ [1]

where *y*_*i*_ is the observed phenotype of each animal i, sex and coat color of each animal *i* are included as fixed effects, *u*_*i*_ represents the random polygenic infinitesimal effect for animal *i* with mean 0 and covariance structure according to the relationships among pedigree members, aSNPi represents the SNP additive fixed effect, dSNPi represents the SNP dominant fixed effect for each SNP and *e*_*i*_ is the random residual. A *p*-value for association was computed for each SNP using the Chi-square approximation to the log-likelihood ratio test (with 2 degrees of freedom). Concomitantly, a Fisher’s exact test, which is robust to the departure from normality and small sample size, was carried out by using R software (www.r-project.org).

A SNP was declared significantly associated to the phenotype if it reached the multiple testing corrected threshold of 5 × 10^–6^. This genome-wide significant threshold was computed using the Bonferroni correction applied to the effective number of independent SNPs tested in the GWAS. The effective number of independent SNPs tested across the genome was estimated by the sum of the effective numbers of independent genotyped SNPs calculated for each chromosome using the M_*eff*_ method [36]. We also reported suggestive associations that met the threshold of 5 × 10^–5^.

### Annotation of the identified loci

An associated region was defined by at least one SNP showing a *p*-value < 5.10^–5^. In many cases, several closely located SNPs showed significant signals and were considered as part of the same region. We further considered these regions and extended the position of the most extreme SNPs by 500 kb on each side to ensure including potential candidate genes that would not be tagged properly by the SNPs of the chip. To annotate the loci, the Biomart tool of Ensembl was used to retrieve automatically the gene content between two borders. However, the annotation of the porcine genome being incomplete, we also included the gene content of the corresponding human regions.

### Comparative mapping and identification of candidate genes

The results obtained in the porcine MeLiM model were compared to the published GWAS results obtained for melanoma risk in humans [16, 25–34]. The loci associated with human melanoma were mapped to the porcine genome, and coordinates were extended by 500 kb on either side of the loci. Thus, ∼1Mb regions were identified in the porcine genome and the information relative to the SNPs located in the interval was retrieved for the 3 MeLiM phenotypes, using results obtained with the Fisher’s exact test. If variants evidenced in human studies were located in intergenic regions, the extension to 1Mb region allowed a proper coverage. Also, the large LD blocks expected in our experimental cross warrant the identification of association signals if they exist. Conversely, the regions corresponding to the swine GWAS signals were mapped onto the human physical and cytogenetic maps, using the NARCISSE tool [91]. The comparison was performed with level 2 syntenies.

## SUPPLEMENTARY MATERIALS FIGURE AND TABLES









## Abbreviations

BC: Backcross
GWAS: Genome-Wide Association Study
HSA: *Homo sapiens* chromosome
LD: Linkage Desequilibrium
MAF: Minimum Allele Frequency
MeLiM: Melanoblastoma-bearing Libechov Minipig
QTL: quantitative trait locus
SLA: Swine Leucocyte Antigen system
SNP: Single Nucleotide Polymorphism
SSC: *Sus scrofa* chromosome
