# Supplementary material for: New susceptibility loci for cutaneous melanoma risk and progression revealed using a porcine model
Source: Oncotarget. 2018 Jun 12;9(45):27682–97. doi: 10.18632/oncotarget.25455 (PMC6021234; doi:10.18632/oncotarget.25455)
Supplement: Supplementary file 1 [file oncotarget-09-27682-s001.pdf]

New susceptibility loci for cutaneous melanoma risk and progression revealed using a porcine model

SUPPLEMENTARY MATERIALS

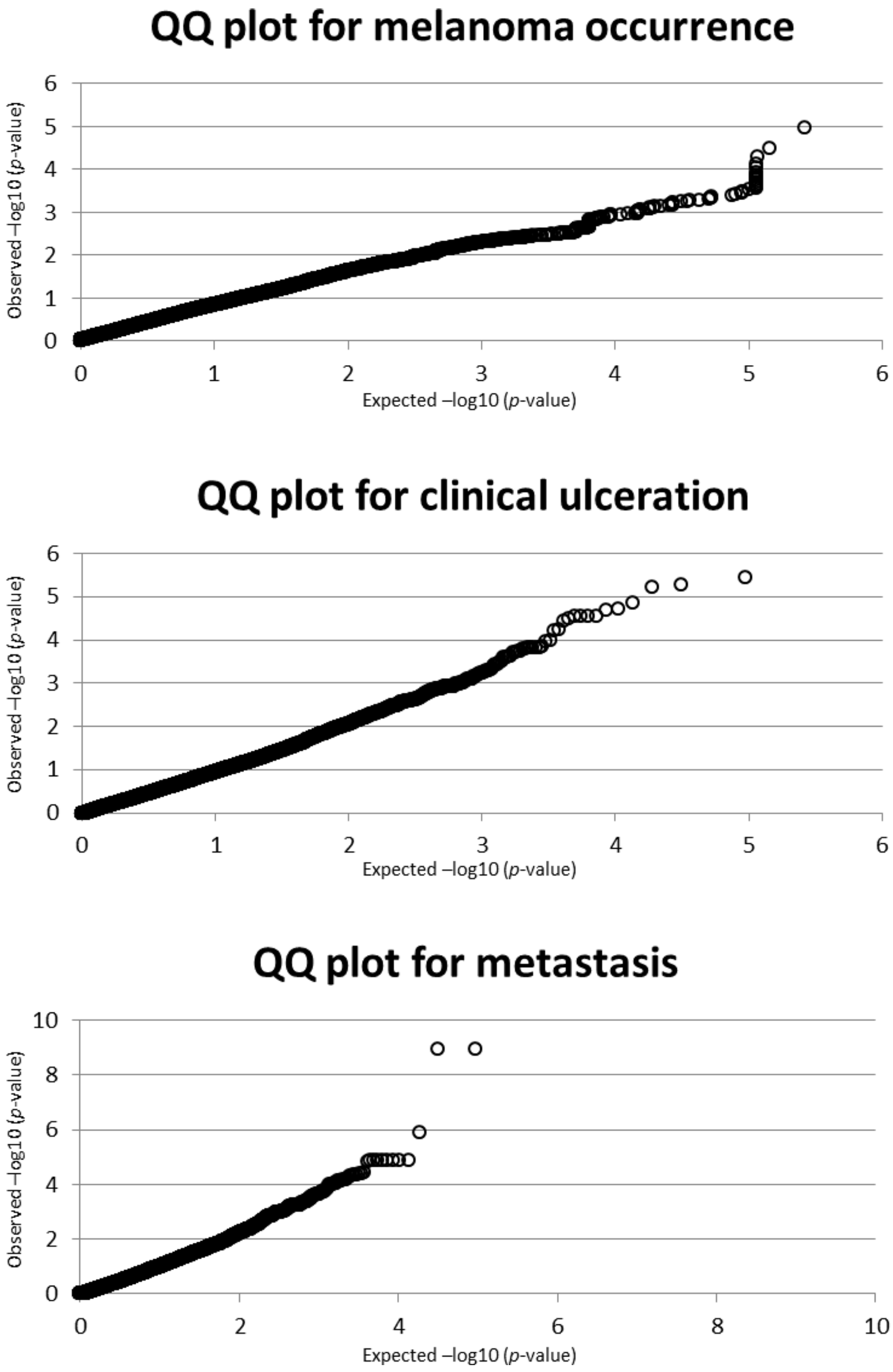

Supplementary Figure 1: QQ-plots obtained for the mixed model analysis of the three phenotypes (melanoma occurrence, clinical ulceration and metastasis).

Supplementary Table 1: Fisher's exact test results obtained for the intervals associated with melanoma occurrence, performed with the mixed model. SNPs are identified by their pig consortium names and rsID. See Supplementary_Table_1

Supplementary Table 2: Fisher's exact test results obtained for the intervals associated with clinical ulceration, performed with the mixed model. See Supplementary_Table_2

Supplementary Table 3: Fisher's exact test results obtained for the intervals associated with metastasis, performed with the mixed model. See Supplementary_Table_3

Supplementary Table 4: Gene content of the genomic regions associated with melanoma occurrence

SSC	Location (bp)	Region size	Number of SNPs	best SNP	Candidate gene	Gene content
**5**	12193129–17986251	5.8 Mb	29	H3GA0015760	NUAK1	SYN3, FBXO7, BPIFC, RTCB, ASCL4, PRDM4, PWP1, BTBD11, CRY1, MTERF2, TMEM263, RIC8B, RFX4, POLR3B, TCP11L2, CKAP4, NUAK1, CCNT1, LINC00935, ADCY6, CACNB3, RND1, CCDC65, FKBP11, WNT10B, WNT1, DDN, PRKAG1, RHEBL1, TUBA1A, DHH, LMBR1L, KMT2D, TUBA1B, PRPH, TROAP, C1QL4, DNAJC22, SPATS2, KCNH3, MCRS1, FAM186B, PRPF40B, FMNL3, AQP6, AQP5, AQP2, FAIM2, BCDIN3D, NCKAP5L, GPD1, SMARCD1, ASIC1, RACGAP1, CERS5, LIMA1, FAM186A, LARP4, DIP2B, ATF1, POU6F1, CELA1, DAZAP2, SMAGP, BIN2, SLC4A8, SCN8A, ANKRD33, ACVRL1, ACVR1B, GRASP
**5**	37633973–38064778	0.4 Mb	6	MARC0071790	TSPAN8	PTPRR, TSPAN8, LGR5, ZFC3H1
**5**	48236817–57199368	8.9 Mb	10	DRGA0005864	PLEKHA5	CCDC91, PTHLH, KLHL42, MANSC4, MRPS35, REP15, PPFIBP1, C12orf71, SMCO2, ARNTL2, STK38L, MED21, TM7SF3, FGFR1OP2, ASUN, ITPR2, SSPN, BHLHE41, RASSF8, LMNTD1, KRAS, ETFRF1, LRMP, BCAT1, SOX5, CMAS, ABCC9, KCNJ8, GYS2, GOLT1B, RECQL, PYROXD1, IAPP, SLCO1A2, SLCO1B1, SLCO1C1, PDE3A, AEBP2, PLEKHA5
**14**	42057436–45642051	3.5 Mb	3	BGIS0007278	TRAFD1	TRAFD1, NAA25, ERP29, TMEM116, MAPKAPK5, ALDH2, BICDL1, PLA2G1B, RAB35, GCN1, MSI1, SIRT4, COX6A1, TRIAP1, GATC, SRSF9, COQ5, DYNLL1, RNF10, POP5, CABP1, MLEC, UNC119B, ACADS, SPPL3, HNF1A, C12orf43, OASL, ANKRD13A, GIT2, TCHP, GLTP, TRPV4, FAM222A, MVK, MMAB, UBE3B, KCTD10, MYO1H, FOXN4, ACACB, UNG, ALKBH2, USP30, SVOP, DAO, SSH1, SELPLG, TMEM119, ISCU, SART3, FICD, CMKLR1, WSCD2, PIWIL3, SGSM1
**14**	51144139§	-	1	DIAS0004694	LIMK2	DUSP18, C5orf52, OSBP2, MORC2, SMTN, SELENOM, INPP5J, PLA2G3, RNF185, LIMK2, PIK3IP1, PATZ1, DRG1, EIF4ENIF1, PISD, SFI1, PRR14L
**15**	141923807§	-	1	ASGA0071118	IRS1	RHBDD1, IRS1, COL4A4

Loci defined by single SNPs (noted as §) have been extended to 1Mb intervals, by adding 500 kb on each side of the significant SNP position.

Supplementary Table 5: Gene content of the genomic regions associated with clinical ulceration

SSC	Location (bp)	Region size	Number of SNPs	Best SNP	Candidate gene	Gene content
**2**	21891536§	-	1	MARC0015434	API5*	-
**5**	14863057§	-	1	ALGA0030768	NUAK1	NUAK1, CCNT1, LINC00935, ADCY6, CACNB3, RND1, CCDC65
**7**	16354696–16407894§	<0.1 Mb	2	ASGA0031451	ID4	ID4, MBOAT1, E2F3, CDKAL1
**7**	33558872–33876748§	0.3 Mb	5	ALGA0040113	DST	ZBTB22, DAXX, KIFC1, BAK1, DST, COL21A1, VPS52, RPS18, B3GALT4, WDR46, PFDN6, RGL2, TAPBP
**7**	123301941§	-	1	ALGA0045159	DICER1	UABP-2, SERPINA12, SERPINA5, SERPINA3, GSC, DICER1, CLMN
**10**	67879073§	-	1	ALGA0115327	CELF2*	-
**13**	6211135–6304167§	0.1 Mb	2	DRGA0011892	SATB1	SATB1
**16**	85304231§	-	1	ASGA0074817	IRX4	IRX4, MRPL36, NDUFS6, LPCAT1

Loci defined by single SNPs or spanning a small region (labeled with §) have been extended to 1 Mb intervals, by adding 500 kb on each side of the significant SNP position. *API5 and CELF2 genes are located more than 1 Mb away from the best SNP.

Supplementary Table 6: Gene content of the genomic regions associated with metastasis

SSC	Location (bp)	Region size	Number of SNPs	Best SNP	Gene closest to best SNP	Gene contenta
**1**	192923645§	-	1	DRGA0001676	LRFN5*	-
**1**	262085978–263273355	1.1 Mb	3	ASGA0006090	SPATA31D1	SPATA31D1, FAM205A
**2**	119655674–121833541	2.1 Mb	2	M1GA0003057	EPB41L4A-AS2	TMEM232, SLC25A46, TSLP, WDR36, CAMK4, STARD4, MCC, DCP2, REEP5, SRP19, APC, EPB41L4A-AS2, EPB41L4A-AS1, EPB41L4A
**5**	6499866–6793026§	0.3 Mb	5	ALGA0030187	CBY1	SYNGR1, RPL3, PDGFB, CBX7, APOBEC3H, NPTXR, DNAL4, SUN2, GTPBP1, JOSD1, TOMM22, CBY1, DMC1, DDX17, KDELR3, KCNJ4, CSNK1E, TMEM184B, MAFF, PLA2G6
**7**	15842505–16370134§	0.5 Mb	3	M1GA0009643	ID4	ID4, MBOAT1
**8**	139895592–139896949§	<0.1 Mb	2	ALGA0114256	HERC3	FAM13A, HERC3, NAP1L5, PYURF, HERC5, ABCG2, PKD2, SPP1
**13**	133415925–134127955§	0.7 Mb	5	MARC0004732	ETV5	ETV5, DGKG, CRYGS, TBCCD1, AHSG, FETUB, HRG, KNG1, EIF4A2, RFC4
**14**	81218453–87459151	6.2 Mb	11	ASGA0064587	USP54	DNAJB12, MICU1, MCU, OIT3, PLA2G12B, P4HA1, FAM149B1, ECD, NUDT13, FAM149B1, DNAJC9, MRPS16, CFAP70, ANXA7, MSS51, PPP3CB, USP54, MYOZ1, SYNPO2L, SEC24C, FUT11, CHCHD1, ZSWIM8, NDST2, CAMK2G, PLAU, VCL, POLR3A, AP3M1, KAT6B, DUPD1, DUSP13, SAMD8, ZNF503, DLG5, VCL, ADK

Loci defined by single SNPs or spanning a small region (labeled with [§]) have been extended to 1 Mb intervals, by adding 500 kb on each side of the significant SNP position. *LRFN5 gene is located more than 1 Mb away from the best SNP DRGA0001676.

Supplementary Table 7: Detailed location of enlarged porcine GWAS peaks on the corresponding human loci, on physical and cytogenetic maps

SSC	Location (bp)	Melanoma Occurrence	Ulceration	Metastasis	Position on the human genome (GRCh37)	Position on the cytogenetic map (GRCh37)
**1**	192923645			√	14:43100000–44300000	14q21.2
**1**	262085978–263273355			√	9:84600000–85000000; 9:85400000–85200000 (–1); no homology from 262750000	9q21.33
**2**	21891536		√		11: 42400000–41300000 (–1)	11p12
**2**	119655674–121833541			√	5: 109500000–110900000; 5:112900000–111300000 (–1)	5q21.3–22.2
**5**	48236817–57199368	√			12:30100000–28350000 (–1); 12:28200000–28300000; 12:28040000–26400000 (–1); 12:26300000–22800000 (–1); 12:22300000–20800000 (–1); 12:20700000–19200000 (–1)	12p12.3–p11.23
**5**	37633973–38064778	√			12:70200000–72800000	12q15–21.1
**5**	12193129–17986251	√	√		22:33000000–32800000 (–1); 12:108200000–106800000 (–1); 12:49080000–50050000; 12:50380000–50180000 (–1); 12:51600000–52400000	22q12.3; 12q23.3; 12q13.12–13
**5**	6499866–6793026			√	22:39100000–38820000 (–1)	22q13.1
**7**	122582935–123664572		√		14:94700000–95650000	14q32.13
**7**	33558872–33876748		√		6:56550000–56150000 (–1)	6p12.1
**7**	15842505–16407894		√	√	6:19450000–20070000	6p22.3
**8**	139895592–139896949			√	4: 89618500–89617000 (–1)	4q22.1
**10**	67879073		√		10:10700000–9500000 (–1)	10p14
**13**	6211135–6304167		√		3:18550000–18650000	3p24.3
**13**	133415925–134127955			√	3:185900000–186650000	3q27.3
**14**	81218453–87459151			√	10:74000000–78000000; 10:78500000–80500000	10q22.1–22.3
**14**	42057436–45642051	√			12:112700000–112200000 (–1); 12:110400000–108300000 (–1)	12q24.12–13; 12q23.3–24.11
**14**	51144139	√			22:31100000–32250000	22q12.2
**15**	141923807		√		2: 227100000–227950000	2q36.3
**16**	85304231		√		5:1050000–1800000 (–1)	5p15.33

(−1) corresponds to an inverted synteny block.

Supplementary Table 8: Summary of the genes identified with the GWAS analysis and located closest to association signals

Gene	Phenotype			Literature data	Involvement in human melanoma
	Melanoma Occurrence	Tumor ulceration	Metastasis		
CBY1			*	Antagonist of β-catenin, key component in melanoma signaling	
CKAP4	***	*	*	Tumor progression	
DICER1		*		Regulated expression in melanoma	X
DST		*		Keratinocyte integrity	X
EPB41L4A-AS2			***	Tumor suppressor	
ETV5			*	Cytokine production, migration of tumor cells	
HERC3			***	Regulation of immune response through NF-κb	
ID4		*		Phenotypic switch in melanoma, tumor regression	X
IRS1	*			IGF1R signaling	
IRX4		***		Prostate cancer risk	
LIMK2	*			Keratinocyte adhesion	
NUAK1	***	*	*	Survival, Invasion	X
PLEKHA5	*			Invasion	X
PPPC3B-AS			*	Overexpression in metastatic pancreas cancer	
PTPRR	*			MAPK pathway regulation	
SATB1		*		Regulated expression in melanoma, proliferation, invasion	X
TRAFD1	*			TLR signaling regulation	
TSPAN8	*			Invasion	X

For relevant references, please refer to the main text. *Corresponds to a suggestive association signal, while ***corresponds to a significant association signal, with above mentioned phenotypes.
