# Supplementary material for: New susceptibility loci for cutaneous melanoma risk and progression revealed using a porcine model
Source: Oncotarget. 2018 Jun 12;9(45):27682–97. doi: 10.18632/oncotarget.25455 (PMC6021234; doi:10.18632/oncotarget.25455)
Supplement: Supplementary file 2 [file oncotarget-09-27682-s002.docx]

**Supplementary Table 1: Fisher’s exact test results obtained for the intervals associated with melanoma occurrence, performed with the Mixed model. SNPs are identified by their Pig consortium names and rsID**

| **SSC** | **Location (bp)** | **Number of significant or suggestive SNPs** | | **Best SNP** | | **Best SNP position** | | **MAF** | | **Min p-value** | | **SNP annotation** | **Candidate genes** | |
| --- | --- | --- | --- | --- | --- | --- | --- | --- | --- | --- | --- | --- | --- | --- |
|  |  | **Mixed Model** | **Fisher** | **Mixed Model** | **Fisher** | **Mixed Model** | **Fisher** | **Mixed Model** | **Fisher** | **Mixed Model** | **Fisher** |  | **Mixed Model** | **Fisher** |
| 5 | 12950560-15864572 | 22 | 18 | H3GA0015760 | MARC0108436 | 14116142 | 14475144 | 0.11 | 0.21 | 3.81E-06 | 1.26E-05 (2.6E-05 for H3GA0015760) | Intergenic | Between CKAP4 (10kb) and NUAK1 (98kb) | Between NUAK1 (184kb) and CCNT1 (448kb) |
| 5 | 37633973-38064778 | 5 | 2 | MARC0071790 | | 37767528 | | 0.17 | | 3.74E-05 | 3.79E-05 | Intergenic | Between PTPRR (121kb) and TSPAN8 (341kb) | |
| 5 | 55008930-57199368 | 2 | 2 | DRGA0005864 | | 57199368 | | 0.166 | | 1.99E-05 | 2.61E-05 | Intronic | PLEKHA5, intron 2 | |
| 14 | 42057436-42257854 | 2 | 2 | BGIS0007278 | | 42057436 | | 0.158 | | 1.91E-05 | 7.80E-05 | Synonymous coding | TRAFD1, p.Leu313 | |
| 14 | 51144139 | 1 | 0 | DIAS0004694 | | 51144139 | | 0.163 | | 3.98E-05 | 1.01E-04 | Intronic | LIMK2, intron 12 | |
| 15 | 141923807 | 1 | 1 | ASGA0071117 | | 141923807 | | 0.331 | | 1.12E-05 | 6.90E-06 | Intergenic | IRS1 (162kb) | |

In the case of intergenic variants, candidate genes correspond to the two genes surrounding the SNPs and located less than 500kb away. For genes located further than 500 kb, only the closest gene is mentioned.
