# Supplementary material for: New susceptibility loci for cutaneous melanoma risk and progression revealed using a porcine model
Source: Oncotarget. 2018 Jun 12;9(45):27682–97. doi: 10.18632/oncotarget.25455 (PMC6021234; doi:10.18632/oncotarget.25455)
Supplement: Supplementary file 3 [file oncotarget-09-27682-s003.docx]

**Supplementary Table 2: Fisher’s exact test results obtained for the intervals associated with clinical ulceration, performed with the Mixed model**

| **SSC** | **Location (bp)** | **Number of significant or suggestive SNPs** | | **Best SNP** | | | **Best SNP position** | | | **MAF** | | **Min p-value** | | **SNP annotation** | **Candidate genes** | | |
| --- | --- | --- | --- | --- | --- | --- | --- | --- | --- | --- | --- | --- | --- | --- | --- | --- | --- |
|  |  | **Mixed Model** | **Fisher** | **Mixed Model** | **Fisher** | | **Mixed Model** | | **Fisher** | **Mixed Model** | **Fisher** | **Mixed Model** | **Fisher** |  | **Mixed Model** | **Fisher** | |
| 2 | 21891536 | 1 | 0 | MARC0015434 | | | 14116142 | | | 0.266 | | 1.92E-05 | 7.50E-04 | Intergenic | Gene desert | | |
| 5 | 14863057 | 1 | 1 | ALGA0030768 | | | 37767528 | | | 0.191 | | 6.05E-06 | 1.02E-06 | Intergenic | Between CCNT1 and NUAK1 | | |
| 7 | 16354696-16407894 | 2 | 0 | ASGA0031451 | | | 57199368 | | | 0.482 | | 1.37E-05 | 5.48E-04 | Intergenic | ID4 (171kb) | | |
| 7 | 33558872-33876748 | 3 | 0 | ALGA0040113 | | | 42057436 | | | 0.361 | | 2.75E-05 | 1.47E-04 | Intergenic | DST, intron 53 | | |
| 7 | 123301941 | 1 | 1 | ALGA0045159 | | | 51144139 | | | 0.442 | | 5.25E-06 | 8.51E-07 | Intergenic | Between GSC (68kb) and DICER1 (322kb) | | |
| 10 | 67879073 | 1 | 1 | ALGA0115327 | | | 141923807 | | | 0.167 | | 2.86E-05 | 3.41E-05 | Intergenic | Gene desert | | |
| 13 | 6211135-6304167 | 2 | 2 | DRGA0011892 | | MARC0068879 | 6211135 | 6304167 | | 0.132 | 0.133 | 2.07E-05 | 8.41E-06 (1.10E-05 for DRGA0011892) | Intergenic | SATB1 (95kb) | | SATB1 (188kb) |
| 16 | 85304231 | 1 | 1 | ASGA0074817 | | | 85304231 | | | 0.37 | | 3.62E-06 | 1.77E-05 | Intergenic | Between IRX2 (406kb) and IRX4 (254kb) | | |

SNPs are identified by their Pig consortium names and rsID. In the case of intergenic variants, candidate genes correspond to the two genes surrounding the SNPs and located less than 500kb away. For genes located further than 500 kb, only the closest gene is mentioned.
