# Supplementary material for: New susceptibility loci for cutaneous melanoma risk and progression revealed using a porcine model
Source: Oncotarget. 2018 Jun 12;9(45):27682–97. doi: 10.18632/oncotarget.25455 (PMC6021234; doi:10.18632/oncotarget.25455)
Supplement: Supplementary file 4 [file oncotarget-09-27682-s004.docx]

**Supplementary Table 3: Fisher’s exact test results obtained for the intervals associated with metastasis, performed with the Mixed model**

| **SSC** | **Location (bp)** | **Number of significant or suggestive SNPs** | | **Best SNP** | | **Best SNP position** | | **MAF** | | **Min p-value** | | **SNP annotation** | **Candidate genes** | |
| --- | --- | --- | --- | --- | --- | --- | --- | --- | --- | --- | --- | --- | --- | --- |
|  |  | **Mixed Model** | **Fisher** | **Mixed Model** | **Fisher** | **Mixed Model** | **Fisher** | **Mixed Model** | **Fisher** | **Mixed Model** | **Fisher** |  | **Mixed Model** | **Fisher** |
| 1 | 192923645 | 1 | 0 | DRGA0001676 | | 192923645 | | 0.281 | | 4.14E-05 | 1.54E-04 | Intergenic | Gene desert | |
| 1 | 262085978 | 1 | 0 | ASGA0006090 | | 262085978 | | 0.414 | | 3.58E-05 | 5.57E-04 | Synonymous coding | SPATA31D1, Lys1054 | |
| 2 | 121833541 | 1 | 1 | M1GA0003057 | | 121833541 | | 0.095 | | 1.31E-06 | 4.78E-06 | Non coding transcript variant | EPB41L4A-AS2 | |
| 5 | 6499866-6619661 | 2 | 0 | ALGA0030187 | | 6619661 | | 0.263 | | 4.44E-05 | 1.15E-02 | Intergenic | Between CBY1 (17kb) and DMC1 (92kb) | |
| 8 | 139895592-139896949 | 2 | 0 | ALGA0114256 | | 139895592 | | 0.139 | | 1.09E-09 | 5.36E-03 | Intergenic | Between HERC3 (17kb) and PIGY (45kb) | |
| 13 | 133415925-134127955 | 2 | 0 | MARC0004732 | | 133415925 | | 0.172 | | 4.03E-05 | 1.27E-01 | Non synonymous coding (predicted) | ETV5, Tyr271Cys | |
| 14 | 81218453-85991839 | 10 | 0 | ASGA0064587 | | 8243562 | | 0.08 | | 1.37E-05 | 3.79E-04 | Intergenic | Between PPP3CB (1kb) and USP54 (0.9kb) | |

SNPs are identified by their Pig consortium names and rsID.
